# Alternations in morphometric similarity network in mesial temporal epilepsy correlate to neuroinflammatory pathway gene transcriptions

**DOI:** 10.1186/s42494-025-00208-4

**Published:** 2025-03-04

**Authors:** Lu Lu, Chenyang Zhao, Weihao Liao, Peiyu Wang, Yingying Zhang, Dongmei An, Xintong Wu, Hesheng Zhang, Ping Jiang, Yaohui He, Jinpeng Niu, Wei Li, Kangjia Chen, Su Lui, Yu Zhao, Qiyong Gong, Bo Wang, Wei Liao, Josemir W. Sander, Lin Chen, Dong Zhou

**Affiliations:** 1 Department of Neurology, & Institute of Brain Science and Brain-Inspired Technology, West China Hospital, Sichuan University, Chengdu, 610041 Sichuan China; 2 Department of Radiology, West China Hospital, Sichuan University, Sichuan Province, Chengdu, 610041 China; 3 The Clinical Hospital of Chengdu Brain Science Institute, School of Life Science and Technology, University of Electronic Science and Technology of China, Chengdu, 610054 China; 4 State Key Laboratory of Brain and Cognitive Science, Institute of Biophysics, University of the Chinese Academy of Sciences, Beijing, 100101 China; 5 Department of Clinical and Experimental Epilepsy, UCL Queen Square Institute of Neurology, London, WC1N 3BG UK

**Keywords:** Allen Human Brain Atlas, Imaging transcriptome analysis, Hippocampus sclerosis

## Abstract

**Background:**

Mesial temporal lobe epilepsy (mTLE) is the most common form of focal epilepsy, often associated with hippocampal sclerosis. Increasing evidence suggests the pivotal role of neuroinflammation in mTLE onset and progression.

**Methods:**

We used morphometric similarity network (MSN) analysis and the Allen Human Brain Atlas (AHBA) database to investigate structural changes between mTLE and healthy controls, as well as correlation with inflammation-related gene expression.

**Results:**

We identified widespread alterations across the frontal and parietal lobes and cingulate cortex linked to neuroinflammatory genes such as *PRR5, SMAD3*, and *IRF3*. This correlation was even more pronounced in mTLE patients with hippocampal sclerosis compared to those without. Enrichment analysis highlighted pathways related to neurodevelopment and neurodegeneration, supporting a bidirectional link between mTLE and neurodegenerative diseases.

**Conclusions:**

These findings suggest that brain-wide macroscopic morphometric alternations in mTLE are correlated to the neuroinflammation process. It provides circumstantial evidence from a new perspective to support the bidirectional link between mTLE and neurodegenerative diseases.

## Background

Mesial temporal lobe epilepsy (mTLE) is a prevalent form of focal epilepsy characterized by structural atrophy in mesiotemporal regions, particularly the hippocampus [1, 2]. Due to the complex nature of mTLE network disturbances, changes in spatiotemporal regional interconnections and the dynamics of transcription have been linked to the development and prognosis of the disease. Neuroimaging studies have suggested extensive spread of morphological changes in the large-scale neocortex, subcortical atrophy, and multiple temporal and extra-temporal pathways [3]. This understanding has merged the importance of correlating large-scale brain cortical structural differences to brain-wide gene expression and transcription to better understand the extratemporal pathogenic mechanisms.

Morphometric similarity network (MSN) analysis has been used in neuropsychology networks to show the microscale cortical organization [3–6]. The main advantage of MSN is that it captures the interregional correlation of multiple morphometric features from various modalities of structural MRI data in a single individual. Considered the widespread structural brain progression recently in mTLE [7–10] in various measurement of grey matter, white matter and topological patterns, this technique would be keen to generate these divided features into a more focused pathological network. Previous efforts in drug-resistant epilepsy have shown that this method of network analysis would allow the link of the topology of MSNs and the histological similarity and the cytoarchitectonic classes [3]. However, the direct evaluation of MSN in mTLE is yet to be fully explored.

The Allen Human Brain Atlas (AHBA) provides valuable data on brain-wide gene expression patterns, offering a bridge between brain structure and molecular function. Combining the structural change and gene transcripts alternations, it identified the alternations related to disease onset and development at mTLE's microscale architecture and network view. A previous study applied the AHBA database reported 1580 genes, mainly enriched in neuronal signaling and synaptic function, associated with the dynamic brain states of mTLE [11].

We aim to assess alterations in MSN metrics in people with mTLE compared to healthy controls and correlate these changes with neuroinflammatory gene expression patterns. These observations may help further understand how brain-wide gene expression could validate anatomical differences in mTLE, potentially offering a new perspective on the global pathological mechanisms of mTLE and future therapeutic targets.

## Methods

### Study Population

Participants were recruited from the Epilepsy Clinic of the West China Hospital between September 1, 2021 and August 31, 2024. The diagnosis and lateralization of mTLE were made according to the clinical evaluation, ictal semiology, and EEG findings. The mTLE diagnosis criteria of the classification guideline of the ILAE were followed [2, 12]. The diagnosis of mTLE and hippocampal sclerosis was independently made, reviewed and validated by two epileptologists. included 1) 16 to 65 years, 2) no other neurological disorders, and 3) no extratemporal structural finding in MRI. Age- and sex-matched healthy controls were invited from clinic companions during the same study period.

### Neuroimaging data acquisition

Structural 3D weighted imaging was acquired using a Siemens Trio 3.0 T MRI scanner (Siemens Medical, Erlangen, Germany) at West China Hospital. T1-weighted (T1W) images were obtained with following parameters: repetition time (TR) = 1900 ms, echo time (TE) = 2.5 ms, flip angle = 9°, field of view (FOV) = 256 × 256 mm^2^, matrix size = 256 × 256, voxel size = 1 × 1 × 1 mm^3^, and number of slices = 176.

### Construction of MSN

T1-weighted images were preprocessed in surface-based space using FreeSurfer (v6.0, http://surfer.nmr.mgh.harvard.edu/), which included skull stripping, tissue segmentation, and separation of hemispheres and subcortical structures, and modeling of gray/white matter interfaces and pial surfaces. Data quality measures were set as the participants with the Euler number provided by FreeSurfer lower than −200 [13].

The cortical surfaces were divided into 308 equal-sized regions (~ 500 mm^2^) based on the Desikan Killiany (D–K) Atlas [3, 4, 14]. The D–K atlas was parcellated and mapped onto each participant’s surface to generate individual surface parcellations [4]. A backtracking algorithm was used to minimize the influence of the variability in parcel sizes. For each region, morphometric features from the T1-weighted MRI scan were extracted to construct the MSN value, including cortical thickness (CT), gray matter volume (GM), surface area (SA), Gaussian curvature (GC), and mean curvature (MC), presenting the morphometric features in grey matter and curvature [5]. The above morphometric feature vectors were *z*-normalized across regions to account for variation in value distributions. Pearson’s correlation analysis was then performed on the morphometric feature vector between each paired cortical region, forming a 308 × 308 MSN for each participant [3, 5].

The regional MSN was calculated by summing the weighted correlation coefficients between a specific region and its correlations with all other regions. The comparisons were made using generalized linear model (GLM), with age, sex and the estimated total intracranial volume (eTIV) as covariates. The model was fitted for each region to examine the case–control differences, and the two-sided *t*-statistic (contrast = mTLE ‒ controls) was calculated and extracted. We corrected the statistical significance for multiple comparisons using a false-positive correction, which was specific to multiple exploratory analyses at nodal properties [15]. The significance threshold was set at *P* < (1/N, N = 308). Spatial permutation testing (spin test) was used to correct spatial autocorrelation. To further determine the nature of the morphology changes, the disease course was applied to the MSN for liner correlation in that significant region (s).

### Measuring the regional gene expressions with MSN

Gene expression data from the Allen Human Brain Atlas (AHBA, http://human.brain-map.org) were used to link gene expression patterns with the MSN. The dataset was preprocessed following guidelines to form a weighted gene expression map [16]. The AHBA data were then modified to fit the parcellation atlas of equal size used. We then used the abagen package (version 0.1.3) with default settings to fetch and manipulate the AHBA data [17], which resulted in 15,633 genes. The selected genes were further screened for protein expression [18].

Partial least squares (PLS) correlation was used to measure the case–control regional MSN difference with the spatial distribution of transcriptional activities. In the PLS regression, gene expression data were used as predictor variables for regional changes in MSN [19]. The first component of the partial least squares (PLS1) was defined as the linear combination of gene expression value that showed the strongest correlation with regional variations in MSN. Bootstrap was applied to assess the variability of each gene’s PLS1. The Z scores were then calculated by dividing the weight of each gene by its bootstrap standard error, which were used to rank genes based on their contribution to PLS1 [5]. Regional variations in the MSN gene list were represented when genes showing a false discovery rate (FDR) of 5‰, categorized as either positive (PLS1 +) or negative (PLS1 −).

The neuroinflammation genes from the “Genes characterized by ISH in 1000 gene survey in cortex” were obtained from the AHBA (https://human.brain-map.org). Forty-six previously reported genes were identified and selected [6, 20, 21], including: *MTOR, AKT1, AKT2, AKT3, RPTOR, TSC1, TSC2, RHEB, S6K1, 4E-BP1, RICTOR, SIN1, PRR5, AMPK, PIK3CA, PTEN, TLR4, MYD88, TICAM1, NFKB1, IRAK4, TLR3, IRF3, RIPK1, TLR2, HMGB1, AGER, IL6, IL6R, JAK1, JAK2, JAK3, STAT3, TNF, TNRSF1A, TRADD, CASP8, MAPK8, TGFB1, TGFB2, TGFB3, TGFBR1, SMAD2, SMAD3, SMAD4*.

To investigate the role of the identified genes in the PLS analysis, we validated the overlapping genes out of the 7411 background genes. Then, the correlations between the gene expression and case–control changes in MSN were estimated. Statistical Significance was defined as *P* < 0.05, with FDR correction applied for multiple comparisons.

### Enrichment analysis

Genes identified as PLS1 + (Z > 5) or PLS1 − (Z < − 5) were input into the Metascape platform (https://metascape.org/gp/index.html#/main/step1) for pathway enrichment analysis [22]. A meta-analysis of multiple gene lists was conducted to explore pathways (and pathway clusters) that were either shared between or uniquely associated with specific gene lists, as described previously [4]. Enrichment networks were generated by representing each enriched term as a node and connecting nodes with Kappa similarities greater than 0.3. The resulting network was visualized using Cytoscape.

### Statistics analysis

The Mann–Whitney U test was used to analyze the differences between groups for continuous variables. For categorical variables, the chi-square test was applied to assess group proportions. When comparing the MSN difference between mTLE patients and healthy controls (HC) in the positively identified brain regions, a significance level of *P* < 1/308 was used to correct for multiple comparisons. All other tests were corrected using the FDR method to control for multiple testing effects and ensure the reliability of the results.

## Results

We enrolled 59 individuals with mTLE and 61 controls. Among the mTLE group, 47 (80%) had hippocampal sclerosis (HS). The lateralization of the seizure onset zone (SOZ) was confirmed in 46 individuals, of whom 26 had left-sided mTLE (57%). Demographic information of participants is shown in Table 1. No significant difference in the covariates was detected.

**Table 1:** Demographics characteristics of the study cohort

Variables	HCs (*n* = 61)	TLE (*n* = 59)	*P* value
Age at scan (yrs, median, IQR)	26.5 (24, 28.75)	26 (22.75, 31)	0.155
Sex (female/male)	29/32	29/30	0.787
eTIV^a^	1,522,679 ± 141,807	1,466,670 ± 143,766	0.504
Course of the disease (yrs, median, IQR)	/	6 (3,12)	/
Age of onset (yrs, median, IQR)	/	20 (13,25)	/
Hippocampal sclerosis	/	47 (80.3%)	/

^a^Estimated total intracranial volume

### The change of MSN in mTLE

**Fig. 1:**
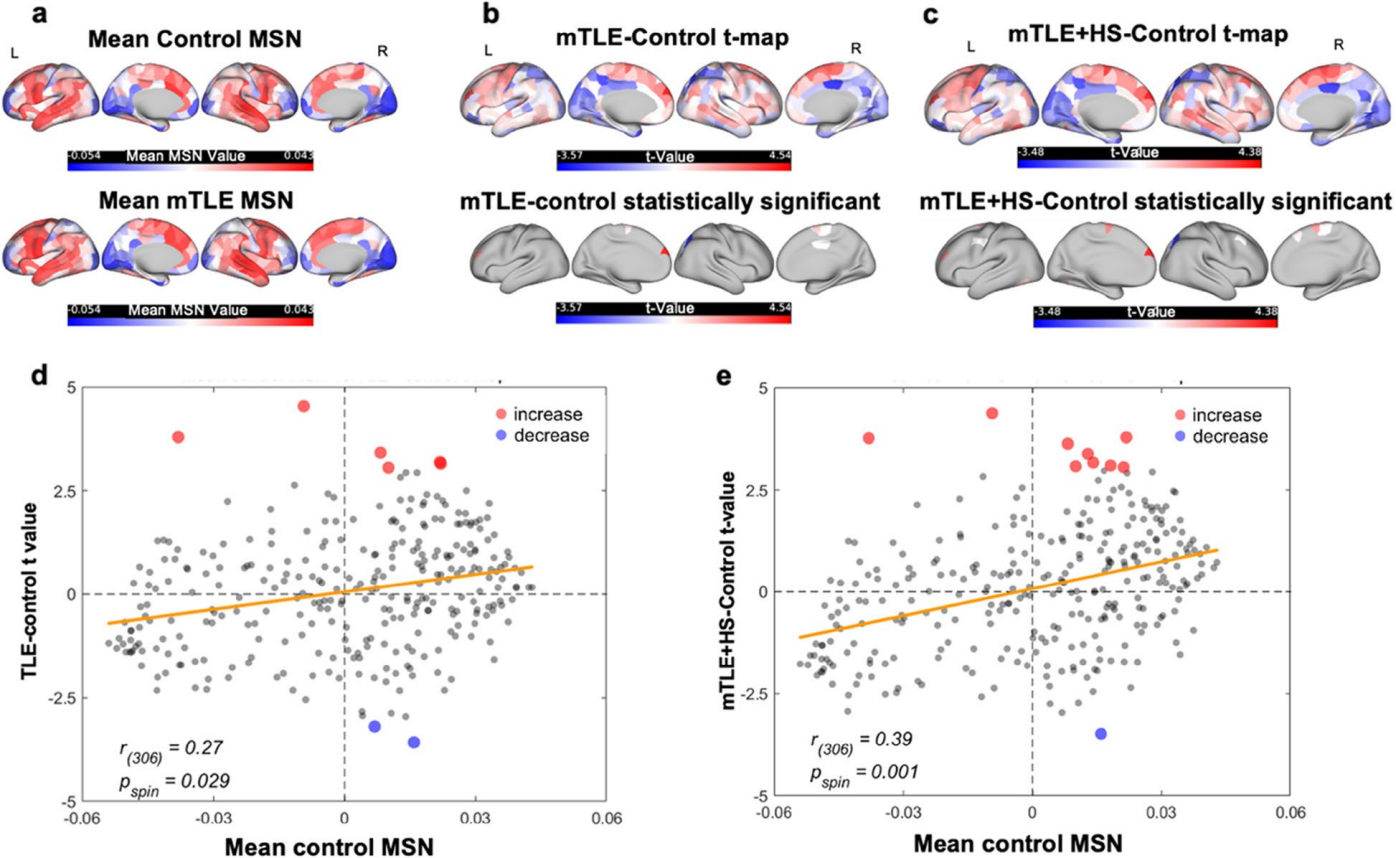
Case–control differences of regional morphometric similarities. **a** The mean regional morphometric similarity network (MSN) pattern of healthy controls (HC) and individuals with mesial temporal lobe epilepsy (mTLE). **b** Case–control comparison (t-map) of MSN between mTLE and HC, eight regions showed significance. **c** Case–control comparison (t-map) of MSN between mTLE with hippocampal sclerosis (HS) and controls, with ten regions showing significant differences. **d** Scatterplot of the mean MSN in control (x-axis) and TLE-HC t value (y-axis) (Pearson’s r_(150)_ = 0.35, *P*_spin_ = 0.004). **e** Scatterplot of the mean MSN in control (x-axis) and t value in TLE withHS (y-axis)

The mean MSN distribution between controls and individuals with mTLE (Fig. 1a) revealed significant differences in eight regions (Fig. 1b). Increased regional MSN in the mTLE group was noted in six extra temporal regions, including lh_superiorfrontal_part5 (t = 4.54, *P* = 1.40E-05), lh_rostralmiddlefrontal_part9 (t = 3.79, *P* = 2.42E-04), rh_superiorfrontal_part3(t = 3.42,*P* = 8.66E-04), lh_superiorfrontal_part2 (t = 3.20,P = 1.79E-03), rh_superiorfrontal_part5 (t = 3.16, *P* = 1.38E-03) and rh_paracentral_part2 (t = 3.06, *P* = 2.77E-03). Two regions showed decreased regional MSN in the mTLE group: rh_superior-parietal_part5 (t = −3.57, *P* = 5.13E-04) and rh_posteriorcingulate_part1(t = −3.19, *P* = 1.83E-03). Increased regional MSN indicates enhanced morphometric similarity and anatomical connection of these regions to the rest of the cortex, conversely for decreased regional MSN. An extra Pearson's correlation analysis was applied to assess the relationship between case–control changes in MSN and the course of the disease. Still, no significant association was found between the regional MSN values in the eight regions where MSN significantly changed between mTLE and controls. A subgroup analysis was performed to compare mTLE, HS, and controls to explore the possible difference, revealing extensive changes in MSNs after FDR correction (Fig. 1c). The extra regions with increased MSN were lh_superiorfrontal_part2 (t = 3.78, *P* = 2.608E-04), lh_fusiform_part5(t = 3.38, *P* = 1.01E-03), rh_superiorfrontal_part11(t = 3.17, *P* = 1.99E-03), lh_precentral_part9 (t = 3.10, *P* = 2.50E-03), rh_caudalmiddlefrontal_part3(t = 3.06, *P* = 2.84E-03). Significant correlations were noted when comparing the subgroups in mTLE with and without HS (Pearson’s r_(150)_, = 0.27, *P*_spin_ = 0.029, Fig. 1e). This correlation was also noted when assessed between the mTLE-HS t map with controls (Pearson’s r_(150)_ = 0.39, *P*_spin_ = 0.001, Fig. 1e) after FDR correction.

### The difference between regional MSN and gene expression

**Fig. 2:**
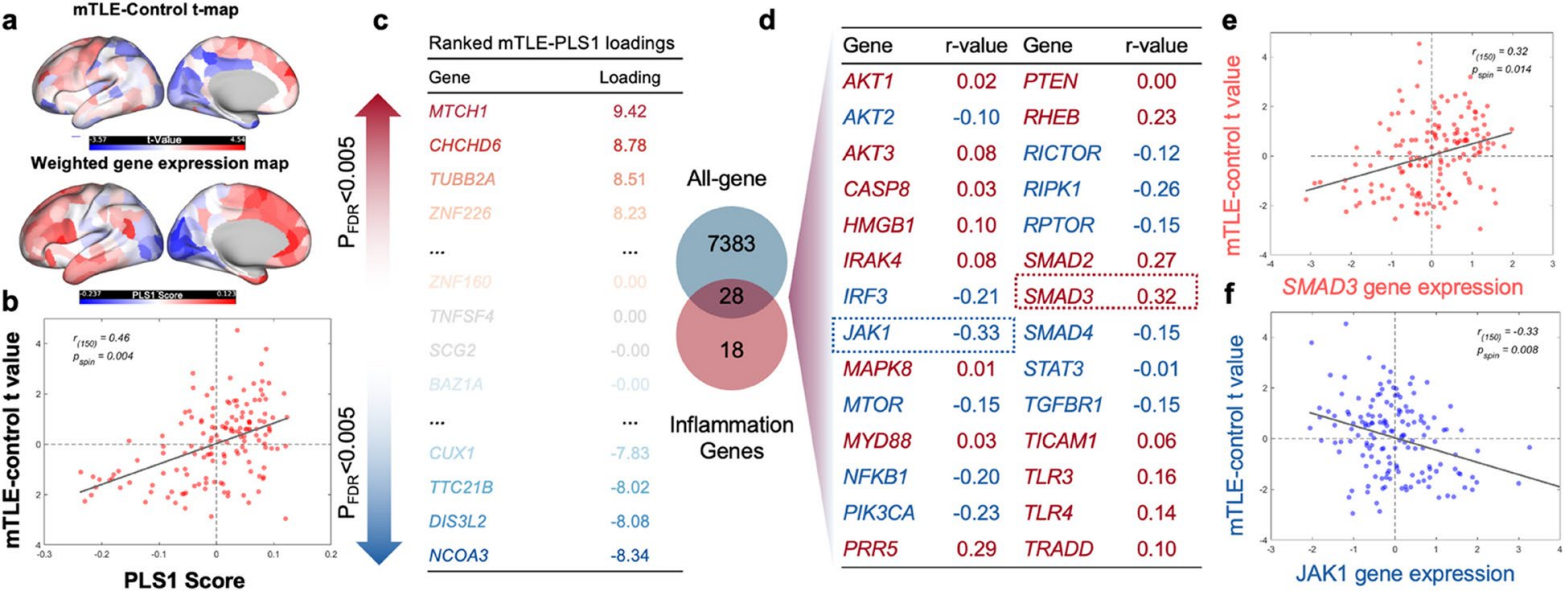
Gene expression profiles related to morphometric similarity differences between mesial temporal lobe epilepsy (mTLE) and healthy controls (HC). **a** Changes in morphometric similarity network (MSN) and the weighted gene expression map of the PLS1 score in the left hemisphere. **b** The scatter plot of the mTLE-HC t value in MSN (y-axis) and the Partial least squares (PLS) 1 score (weighted sum of the 7411 genes), showing Pearson’s r_(150)_ = 0.46, *P*_spin_ = 0.004. **c** Ranked PLS1 loadings in mTLE. **d** Overlap of the selected inflammation genes with all screened genes, where *SMAD3* (Pearson’s r_(150)_ = 0.32, *P*_spin_ = 0.014) and *JAK1* (Pearson’s r_(150)_ = −0.33, *P*_spin_ = 0.008) were significant before FDR correction. **e** Scatterplot of the *SMAD3* gene expression (x-axis) and the mTLE-HC t value (y-axis). **f** Scatterplot of the* JAK1* gene expression (x-axis) and the mTLE-HC t value (y-axis)

The results of the partial least squares regression between regional MSN and gene expression in the left hemisphere are shown in Fig. 2. The first component (PLS1) was the spatial map that captures the most significant fraction of total gene expression variance across cortical areas. The anterior–posterior gradient of gene expression was presented in the weighted gene expression map of the PLS1 score (Fig. 2a). When comparing the weighted gene expression with the MSN, a significant correlation was found between the mTLE-HC t map (Fig. 2b, Pearson’s r_(150)_ = 0.46, *P*_spin_ = 0.004). The normalized weights of PLS1 were ranked based on univariate one-sample Z tests, given 1485 genes that showed significance after correction (*P*_FDR_ < 0.005, Fig. 2c), indicating the over-expression or under-expression of these genes correlated with the increase or decrease in the regional MSN. To determine the relation between these genes and the neuroinflammation process, the pre-defined gene was applied to the AHBA. It resulted in 28 overlapping genes among all genes (Fig. 2d) and two genes with signifcance (Fig. 2e). The gene expression correlation was significant in *SMAD3* (Fig. 2e.) and *JAK1* (Fig. 2f.) in spin test 1000 but failed to show significance after FDR correction.

**Fig. 3:**
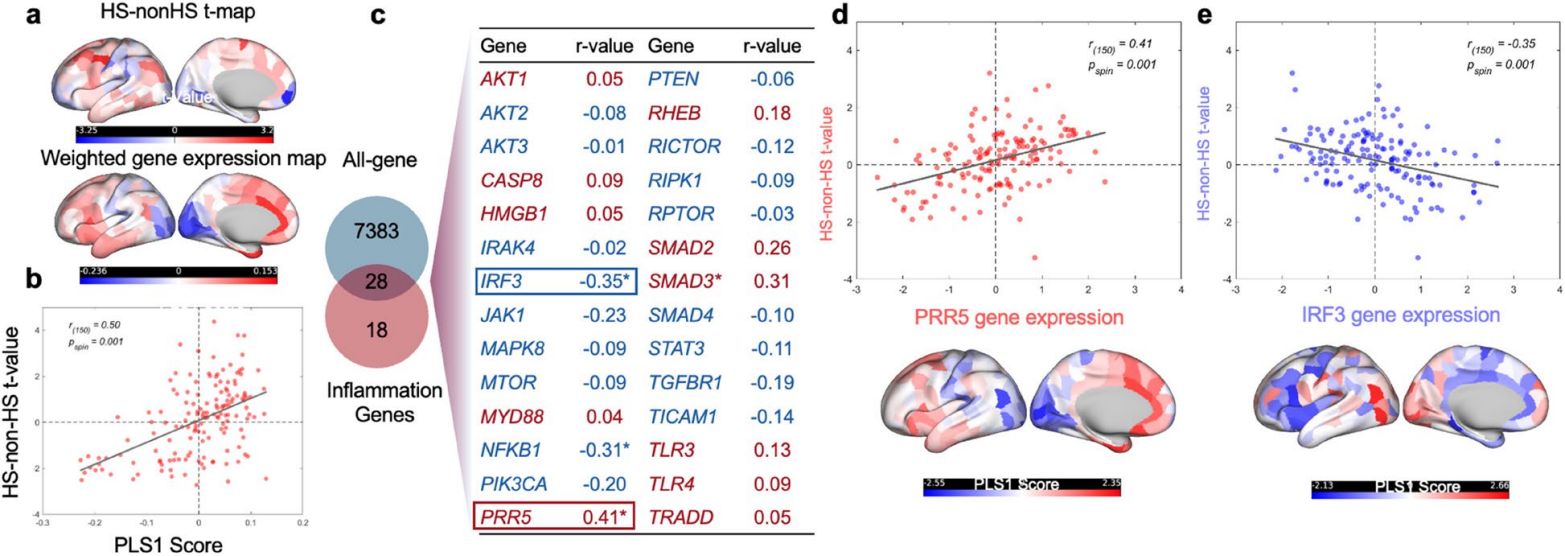
Gene expression profiles related to morphometric similarity (MSN) differences between mesial temporal lobe epilepsy (mTLE) with and without hippocampal sclerosis (HS). **a** Difference in MSN between mTLE with and without HS, along with the weighted gene expression map of the PLS1 score in the left hemisphere. **b** The scatter plot of the HS-non-HS t-value in MSN (y-axis) and the PLS1 score, showing Pearson’s r_(150)_ = 0.50, *P*_spin_ = 0.001. **c** The PLS1 loading and significance in the 28 overlapped genes. (* in *SMAD3* represents significance before FDR correction). Significance was noted in *PRR5* and *IRF3* after FDR correction. **d** Scatter plot of the *PRR5* (positive weighted) gene expression (x-axis) versus the HS-non-HS t-value (y-axis). **e** Scatter plot of *IRF3* (negative weighted) gene expression (x-axis) and the HS-non-HS t-value(y-axis)

The same test was applied to mTLE individuals with HS compared to controls to further explore the gene expression correlation within the control subgroup. The difference in MSN t-value between the mTLE with HS and controls was shown in Fig. 3a, together with the gradient of the weighted gene expression map of the PLS1 score. A significant correlation was found between the difference in MSN of the HS subgroups and the PLS1 scores (*P*earson’s r_(150)_ = 0.49, *P*_spin_ < 0.0001, Fig. 3b). The normalized weights of PLS1 were also ranked based on univariate one-sample Z tests, as shown in Fig. 3c. The 28 overlapping genes were again corrected in FDR, where *IRF3* (*P*earson’s r_(150)_ = −0.31, *P*_spin_ = 0.003)*, SMAD3* (*P*earson’s r_(150)_ = 0.34, *P*_spin_ = 0.003)*, JAK1* (*P*earson’s r_(150)_ = −0.30, *P*_spin_ = 0.003)*, *and* PRR5* (Pearson’s r_(150)_ = 0.35, *P*_spin_ = 0.005), showed significance(Fig. 3d).

### Enrichment pathways associated with changes in MSN

**Fig. 4:**
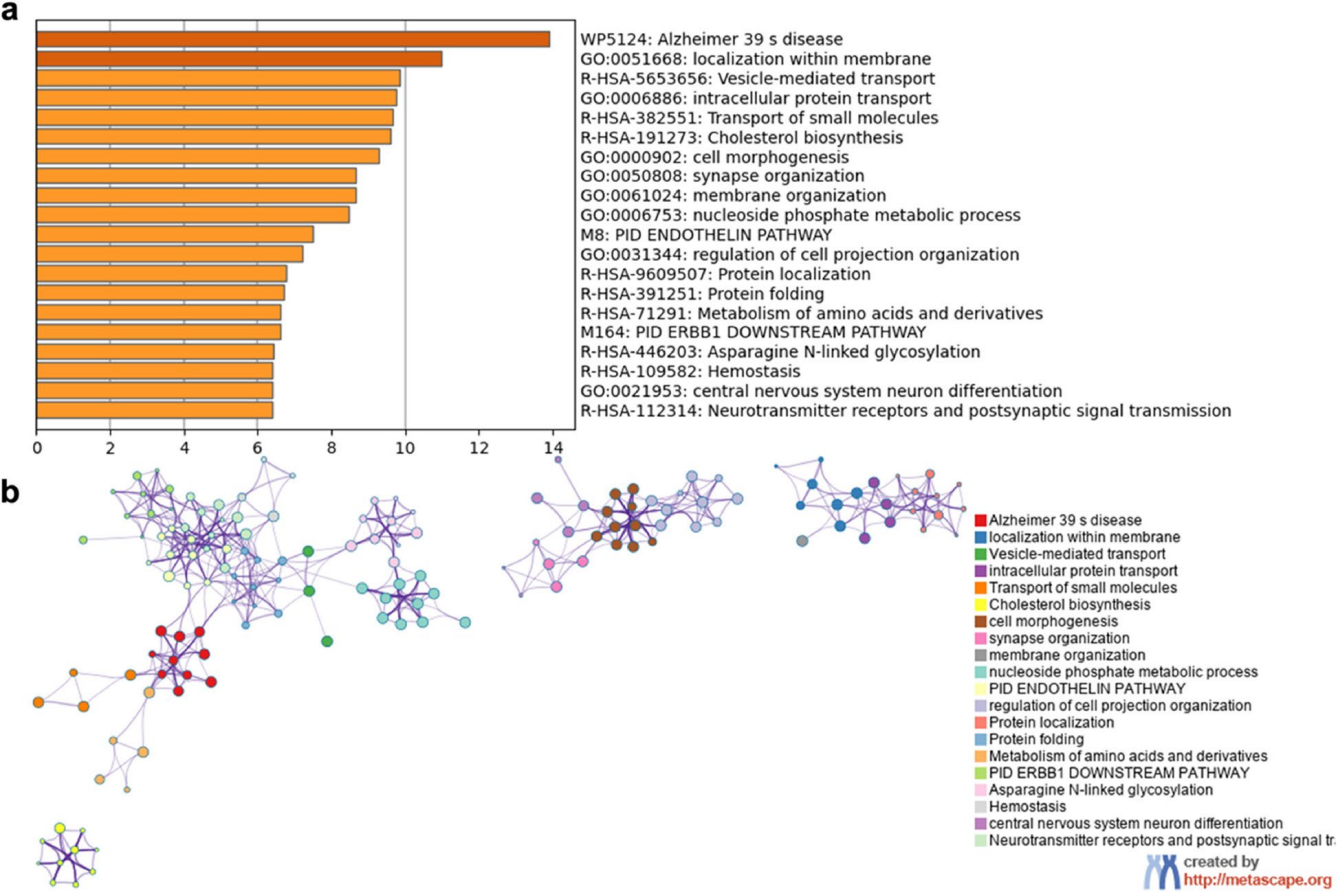
Functional enrichment of gene transcripts. **a** Heatmap of enriched terms across the upregulated genes in mesial temporal lobe epilepsy (mTLE), colored by *P*-values. **b** Metascape enrichment network visualization showing the intra-cluster and inter-cluster similarities of enriched terms

We found that 916 PLS1 + (Z > 5) genes and 827 PLS − (Z < − 5) genes were significantly overexpressed in cortical regions between mTLE and controls. To better summarize the difference in gene transcription, the enrichment pathway analysis results for PLS + genes were shown in Fig. 4a. The top five significantly involved biological processes included Alzheimer’s disease, localization within the membrane, vesicle-mediated transport, intracellular protein transport and transport of small molecules. For visualization, the Metascape enrichment network was presented in Fig. 4b, indicating the similarities of enriched terms within and between clusters.

## Discussion

We assessed alterations in MSN metrics and their correlation with neuroinflammatory gene expression patterns. The results identified widespread, frontal lobe-oriented bilateral morphometric changes in mTLE. These alterations may contribute to disease progression and the development of comorbidities such as Alzheimer’s disease and cognition declines. Additionally, we observed that neuroinflammatory gene expression was spatially correlated with MSN changes, particularly in people with HS. The extra-temporal MSN differences were extensively observed between the mTLE and HS, and gene expression correlations with MSN were more substantial in the HS subgroup. Enrichment analysis of gene pathways showed significant alterations related to Alzheimer’s disease and various domains of cellular transport, aligning with the recent hypothesis that mTLE might be a particular subtype of Alzheimer’s disease. It also examines molecular processes underlying mTLE, with particular emphasis on the role of neuroinflammation in shaping brain networks and influencing cognitive outcomes. These findings provide a more comprehensive understanding of disease progression in mTLE by integrating transcriptomic and neuroimaging changes.

### The MSN changes in mTLE and its global alternation

The widespread structural brain network abnormality in mTLE has been reported in various populations and groups in the measurement of gray matter, white matter and topological patterns, including whole brain volumetric network [7–10]. This pathological progression of mTLE involves widespread neural network remodeling and neuroinflammatory responses. This extratemporal global reorganization has also correlated to continuous progression in mTLE during the disease course, even in those whose seizures are clinically controlled by medications [23]. As knowledge increases regarding the association between the extratemporal structural property changes and possible neuroinflammation and neurodegeneration process [21], the correlation network converging with spatial expression patterns of risk genes for mTLE remains unclear.

 MSN analysis enhanced the understanding of structural brain abnormalities in mTLE by capturing interregional correlations of multiple morphometric features. Unlike traditional methods focusing on single structural measures [23, 24], MSN integrates these structural brain metrics to create a more comprehensive map of structural relationships across regions. The network structure exposed by MSN is highly correlated with distinct cortical regions that share similar laminar patterns and connectivity profiles [3, 6]. Our data suggest significant structural differences between people with mTLE and controls, primarily in regions of the frontal, parietal, and cingulate cortices. Notably, no significant changes were observed in the temporal lobe regions. The six regions with increased MSN observed in mTLE suggested the enhanced morphometric similarity to controls, which might indicate the functional remodeling of neuronal connections within these regions, and the further structural reorganization or excessive activation of these areas in the brain network [4, 6]. In contrast, decreased MSN may indicate increased morphometric differentiation, which could reflect weakened neural connectivity between these areas and other regions of the brain; this weakening could be related to abnormal brain network connectivity or dysfunction, potentially contributing to the progression of the disease. Dysfunction in these regions could be closely associated with cognitive deficits, emotional fluctuations, or behavioral abnormalities in people with mTLE [10, 25].

### Neuroinflammation and gene expression correlation

MSN enables a detailed exploration of how brain areas are interconnected regarding their microstructural properties and the associations among structural network abnormalities, gene expression, and neuroinflammatory processes. By combining MSN with transcriptomic analysis, we found that morphometric alterations were spatially correlated with specific neuroinflammatory genes, such as *SMAD3, IRF3, and PRR5.* A stronger correlation was observed between gene expression and brain network alterations in people with HS. Notably, some studies have shown that mTLE patients may present “hippocampal innate inflammatory gliosis only [26, 27]”, a phenomenon where neuroinflammation is restricted to glial cell proliferation without significant neuronal injury or hippocampal sclerosis. In contrast, mTLE patients with HS exhibit more widespread structural changes and neuroinflammatory responses, which might contribute to the stronger correlations observed between neuroinflammatory gene expression and structural network alterations in this subgroup [28]. The presence of hippocampal sclerosis may therefore drive more extensive reorganization of brain networks and a more pronounced neuroinflammatory response, which could explain the differences in gene expression correlations between these two groups.

These findings suggest that neuroinflammation, possibly through the modulation of these genes, may play a crucial role in driving the structural and functional reorganization of brain networks, especially in non-temporal regions [21, 29].

SMAD3 is a key mediator in the TGF-β signaling pathway. Previous evidence in rodents and human tissue has shown that the level of phospho-Smad3 is significantly upregulated in neurons of the temporal cortex in mTLE compared to controls. It serves as the downstream factor after the activation of TGF-β1 and further activates the transcription of TGF-β/Smad-targeted genes, releases inflammatory cytokines, and triggers or maintains seizure [29–31]. While previous research has mainly focused on temporal lobe tissue, our data suggest that the activation of *SMAD3* could also be triggered at a whole brain scale and might contribute to the overall structural alternation.

Interferon regulatory factor 3 (IRF3) is the downstream mediator of Toll-like receptor 3 (TLR3) that has been previously shown to contribute to the inflammatory process and the generation of epileptic seizures in both directions [32]. Reduced IRF3 signaling could contribute to an environment conducive to neurodegeneration, impaired neurogenesis, and dysfunctional synaptic remodeling, further enhancing the progression of seizures and epilepsy-related changes in the brain [33].

Additionally, we observed significant MSN changes corresponding to increased *PRR5* expression, with more substantial significance observed in subgroup analyses of individuals with and without HS. Proline-rich protein 5 (PRR5) is a component of the mammalian target of rapamycin complex 2 (mTORC2) [34]. Dysregulation of mTORC2 signaling, including the PRR5 component, can contribute to abnormal neuronal growth and reorganization, which may play a role in the development of epilepsy and its progression. Activation of the mTOR pathway and markers of neuroinflammation have been detected in the epileptic foci of people with mTLE and HS.

The transcription results in the MSN changes have noted the involvement of three main neuroinflammation pathways in the whole brain scale. Previous studies [24] have shown that TLE with HS is closely associated with progressive cortical atrophy. Research indicates that people with mTLE experience cortical thinning at a rate that exceeds normal aging, with younger individuals (under 55 years old) showing an annual cortical thinning rate twice that of normal aging, while those older than 55 have an almost four-fold increase in thinning rate. The network pathology of mTLE also reveals that epileptic activity is not confined to traditional foci but extends to broader regions of the brain, particularly affecting "rich club" brain regions, which plays a crucial role in seizure generalization [35]. The overall network topology in people with mTLE is reduced compared to controls, and those with focal seizures are more likely to damage non-"rich club" brain regions. In contrast, those with generalized seizures predominantly damage the "rich club" regions, highlighting the contribution of these core regions to the spread of mTLE seizures. Our transcription results in the MSN changes show that morphological and anatomical connection changes in people with TLE closely correlate with the expression of specific neuroinflammatory genes, particularly in those with HS. These findings suggest that neuroinflammation, possibly through the modulation of these genes, may play a key role in driving the structural and functional reorganization of brain networks. The activation of inflammation-related genes may be one of the critical factors in the pathological progression of mTLE. By further assessing these mechanisms, we could better understand the progression of mTLE and its association with comorbidities such as cognitive decline and mood disorder. Especially in relation to genes associated with the mTOR pathway, considering the approved targeted therapies for tuberous sclerosis [36], future gene therapies [37] targeting these genes or their pathways could offer new strategies for disease-modifying treatments in mTLE.

By capturing the morphological associations between brain regions, we provide new insights into the global changes in brain structural networks, building upon previous findings of widespread brain atrophy in mTLE patients [24]. Several studies have previously explored the relationship between imaging and gene expression in TLE. Qin et al. [11] primarily analyzed the correlation between dynamic functional connectivity patterns and gene expression, finding that in TLE patients with cognitive impairment, changes in dynamic connectivity were closely linked to adjustments in brain network modules, demonstrating a strong genetic dependence. Xiao et al. [38] combined [^18^F]FDG PET imaging and transcriptomic data to examine brain metabolic networks, revealing that metabolic alterations in TLE patients were spatially correlated with the expression of genes related to neurovascular unit integrity and synaptic plasticity. It showed unique spatial distributions of metabolic changes in TLE, which were significantly associated with the expression of genes involved in neuronal function. Li et al. [39] combined [^18^F]SynVesT-1 PET imaging with transcriptomic data to demonstrate dysfunction in the synaptic density similarity network (SDSN) in TLE patients, associated with downregulation of GABAergic genes. This finding intersects with the inflammatory genes and pathways we focus on, suggesting that future research should intensify the study of gene pathways such as neuroinflammation and synaptic transmission in the structural and functional reorganization of the brain in TLE patients.

### Enrichment analysis and pathological link to neurodegenerative diseases

The enrichment analysis exposed significant associations with pathways related to Alzheimer’s disease and transport-related protein expression, underscoring the crucial roles these processes play in the pathophysiology of mTLE. These findings highlight the involvement of these pathology pathways in the progression of mTLE and provide insight into potential common mechanisms linking mTLE to neurodegenerative diseases.

mTLE has been increasingly recognized as a disorder that shares common pathological features with neurodegenerative diseases, particularly Alzheimer's disease [40]. A structural MRI study about the progressive cortical atrophy in mTLE found it was similar to mild cognition impairments [40]. The same cortical and hippocampal atrophy patterns in both conditions have correlated with the development of cognitive dysfunction, but not with the course of epilepsy [41]. The dysregulation of neurodevelopmental pathways in mTLE is critical to the progression of the disease. Neuroinflammation, as observed in mTLE, significantly impacts neuronal development and synaptic plasticity, which are essential for cognitive functions. This disruption may lead to cognitive deficits in mTLE, a phenomenon that mirrors similar disruptions observed in neurodegenerative diseases such as Alzheimer’s disease.

We found that individuals with mTLE exhibited significant alterations in MSN, particularly in the frontal, parietal, and cingulate cortex. These changes were associated with the expression of neuroinflammatory genes, such as *PRR5, SMAD3*, and *IRF3*, especially in those with hippocampal sclerosis. The most prominent pathways identified in the enrichment analysis were nervous system development and neurodegenerations. These findings underscore the role of neuroinflammation in the reorganization of brain networks in mTLE, suggesting that neuroinflammatory processes may contribute to progressive structural and functional alterations in the brain. It supports the potential for early intervention in mTLE to prevent or slow down the progression of neurodegeneration behind the inflammation.

However, this study presents several limitations that should be considered. Firstly, the single-center retrospective cohort with a limited sample size might restrict the effect and stability of the current results. Secondly, as with the previous studies that applied the AHBA, the right hemisphere was not selected due to the limitations in the availability of correct hemisphere data. Thirdly, previous studies have reported similar effects in 5- features MSN to 10- features MSN, but more features could be applied to draft the MSN in TLE as validation.

## Conclusions

This study identifies widespread structural abnormalities in mTLE, particularly in the frontal, parietal, and cingulate cortices, with significant correlations to neuroinflammatory gene expression. These findings support a bidirectional link between mTLE and neurodegenerative diseases, especially in patients with hippocampal sclerosis. By integrating MSN analysis with transcriptomic data, we provide a novel perspective on how brain-wide structural changes are driven by neuroinflammation, suggesting new biomarkers and potential therapeutic targets for modifying early progression of mTLE.

## Data Availability

The data that support the findings of this study are available from the corresponding author upon reasonable request.

## Abbreviations

AD: Alzheimer's disease
AHBA: Allen Human Brain Atlas
CT: Cortical thickness
FDR: False discovery rate
eTIV: Estimated total intracranial volume
GM: Gray matter volume
GLM: Generalized linear model
GC: Gaussian curvature
HS: Hippocampal sclerosis
IRF3: Interferon regulatory factor 3
mTLE: Mesial temporal lobe epilepsy
MC: Mean curvature
mTOR: Mammalian target of Rapamycin
mTORC2: Mammalian target of Rapamycin complex 2
MSN: Morphometric similarity network
PRR5: Proline-rich protein 5
PLS: Partial least squares
PLS1: First component of partial least squares
SA: Surface area
SMAD3: SMAD family member 3
Spin: Spatial permutation test
T1W: T1-weighted
TGF-β: Transforming growth factor beta

## References

[1] Jiang Y, Li W, Li J, Li X, Zhang H, Sima X, et al. Identification of four biotypes in temporal lobe epilepsy via machine learning on brain images. Nat Commun. 2024;15(1):2221. 38472252 10.1038/s41467-024-46629-6 PMC10933450

[2] Blümcke I, Thom M, Aronica E, Armstrong DD, Bartolomei F, Bernasconi A, et al. International consensus classification of hippocampal sclerosis in temporal lobe epilepsy: a Task Force report from the ILAE Commission on Diagnostic Methods. Epilepsia. 2013;54(7):1315–29. 23692496 10.1111/epi.12220

[3] Seidlitz J, Váša F, Shinn M, Romero-Garcia R, Whitaker KJ, Vértes PE, et al. Morphometric similarity networks detect microscale cortical organization and predict inter-individual cognitive variation. Neuron. 2018;97(1):231-247.e7. 29276055 10.1016/j.neuron.2017.11.039 PMC5763517

[4] Li J, Seidlitz J, Suckling J, Fan F, Ji GJ, Meng Y, et al. Cortical structural differences in major depressive disorder correlate with cell type-specific transcriptional signatures. Nat Commun. 2021;12(1):1647. 33712584 10.1038/s41467-021-21943-5 PMC7955076

[5] Morgan SE, Seidlitz J, Whitaker KJ, Romero-Garcia R, Clifton NE, Scarpazza C, et al. Cortical patterning of abnormal morphometric similarity in psychosis is associated with brain expression of schizophrenia-related genes. Proc Natl Acad Sci U S A. 2019;116(19):9604–9. 31004051 10.1073/pnas.1820754116 PMC6511038

[6] Cao H, Wei P, Huang Y, Wang N, Guo LA, Fan X, et al. The alteration of cortical microstructure similarity in drug-resistant epilepsy correlated with mTOR pathway genes. eBioMedicine. 2023;97:104847. 39492369 10.1016/j.ebiom.2023.104847 PMC10628344

[7] Larivière S, Royer J, Rodríguez-Cruces R, Paquola C, Caligiuri ME, Gambardella A, et al. Structural network alterations in focal and generalized epilepsy assessed in a worldwide ENIGMA study follow axes of epilepsy risk gene expression. Nat Commun. 2022;13(1):4320. 35896547 10.1038/s41467-022-31730-5 PMC9329287

[8] Yasuda CL, Chen Z, Beltramini GC, Coan AC, Morita ME, Kubota B, et al. Aberrant topological patterns of brain structural network in temporal lobe epilepsy. Epilepsia. 2015;56(12):1992–2002. 26530395 10.1111/epi.13225

[9] DeSalvo MN, Douw L, Tanaka N, Reinsberger C, Stufflebeam SM. Altered structural connectome in temporal lobe epilepsy. Radiology. 2014;270(3):842–8. 24475828 10.1148/radiol.13131044 PMC4263659

[10] Sinha N, Peternell N, Schroeder GM, de Tisi J, Vos SB, Winston GP, et al. Focal to bilateral tonic-clonic seizures are associated with widespread network abnormality in temporal lobe epilepsy. Epilepsia. 2021;62(3):729–41. 33476430 10.1111/epi.16819 PMC8600951

[11] Qin L, Zhou Q, Sun Y, Pang X, Chen Z, Zheng J. Dynamic functional connectivity and gene expression correlates in temporal lobe epilepsy: insights from hidden markov models. J Transl Med. 2024;22(1):763. 39143498 10.1186/s12967-024-05580-2 PMC11323657

[12] Fisher RS, Cross JH, French JA, Higurashi N, Hirsch E, Jansen FE, et al. Operational classification of seizure types by the International League Against Epilepsy: Position Paper of the ILAE Commission for Classification and Terminology. Epilepsia. 2017;58(4):522–30. 28276060 10.1111/epi.13670

[13] Rosen AFG, Roalf DR, Ruparel K, Blake J, Seelaus K, Villa LP, et al. Quantitative assessment of structural image quality. Neuroimage. 2018;1(169):407–18. 10.1016/j.neuroimage.2017.12.059 PMC5856621 29278774

[14] Romero-Garcia R, Atienza M, Clemmensen LH, Cantero JL. Effects of network resolution on topological properties of human neocortex. Neuroimage. 2012;59(4):3522–32. 22094643 10.1016/j.neuroimage.2011.10.086

[15] Liao W, Li J, Duan X, Cui Q, Chen H, Chen H. Static and dynamic connectomics differentiate between depressed patients with and without suicidal ideation. Hum Brain Mapp. 2018;39(10):4105–18. 29962025 10.1002/hbm.24235 PMC6866497

[16] Arnatkeviciute A, Fulcher BD, Fornito A. A practical guide to linking brain-wide gene expression and neuroimaging data. Neuroimage. 2019;1(189):353–67. 10.1016/j.neuroimage.2019.01.011 30648605

[17] Markello RD, Arnatkeviciute A, Poline JB, Fulcher BD, Fornito A, Misic B. Standardizing workflows in imaging transcriptomics with the abagen toolbox. Elife. 2021;16(10):e72129. 10.7554/eLife.72129 PMC8660024 34783653

[18] Shanmugan S, Seidlitz J, Cui Z, Adebimpe A, Bassett DS, Bertolero MA, et al. Sex differences in the functional topography of association networks in youth. Proc Natl Acad Sci U S A. 2022;119(33):e2110416119. 35939696 10.1073/pnas.2110416119 PMC9388107

[19] Abdi H, Williams LJ. Partial least squares methods: partial least squares correlation and partial least square regression. Methods Mol Biol. 2013;930:549–79. 23086857 10.1007/978-1-62703-059-5_23

[20] Soltani Khaboushan A, Yazdanpanah N, Rezaei N. Neuroinflammation and Proinflammatory Cytokines in Epileptogenesis. Mol Neurobiol. 2022;59(3):1724–43. 35015252 10.1007/s12035-022-02725-6

[21] Ravizza T, Scheper M, Di Sapia R, Gorter J, Aronica E, Vezzani A. mTOR and neuroinflammation in epilepsy: implications for disease progression and treatment. Nat Rev Neurosci. 2024;25(5):334–50. 38531962 10.1038/s41583-024-00805-1

[22] Zhou Y, Zhou B, Pache L, Chang M, Khodabakhshi AH, Tanaseichuk O, et al. Metascape provides a biologist-oriented resource for the analysis of systems-level datasets. Nat Commun. 2019;10(1):1523. 30944313 10.1038/s41467-019-09234-6 PMC6447622

[23] Sone D, Beheshti I, Maikusa N, Ota M, Kimura Y, Sato N, et al. Neuroimaging-based brain-age prediction in diverse forms of epilepsy: a signature of psychosis and beyond. Mol Psychiatry. 2021;26(3):825–34. 31160692 10.1038/s41380-019-0446-9 PMC7910210

[24] Galovic M, van Dooren VQH, Postma TS, Vos SB, Caciagli L, Borzì G, et al. Progressive Cortical Thinning in Patients With Focal Epilepsy. JAMA Neurol. 2019;76(10):1230–9. 31260004 10.1001/jamaneurol.2019.1708 PMC6604082

[25] Stretton J, Pope RA, Winston GP, Sidhu MK, Symms M, Duncan JS, et al. Temporal lobe epilepsy and affective disorders: the role of the subgenual anterior cingulate cortex. J Neurol Neurosurg Psychiatry. 2015;86(2):144–51. 24876189 10.1136/jnnp-2013-306966 PMC4316913

[26] Grote A, Heiland DH, Taube J, Helmstaedter C, Ravi VM, Will P, et al. “Hippocampal innate inflammatory gliosis only” in pharmacoresistant temporal lobe epilepsy. Brain. 2023;146(2):549–60. 35978480 10.1093/brain/awac293 PMC9924906

[27] Taube J, Witt J, Grote A, et al. Preoperative and postoperative memory in epilepsy patients with ‘gliosis only’ versus hippocampal sclerosis: a matched case–control study. J Neurol Neurosurg Psychiatry. 2022;93:1202–8. 10.1136/jnnp-2022-329224 36008114

[28] Aulická S, Česká K, Šána J, Siegl F, Brichtová E, Ošlejšková H, et al. Cytokine-chemokine profiles in the hippocampus of patients with mesial temporal lobe epilepsy and hippocampal sclerosis. Epilepsy Res. 2022;180:106858. 35026708 10.1016/j.eplepsyres.2022.106858

[29] Vezzani A, Balosso S, Ravizza T. Neuroinflammatory pathways as treatment targets and biomarkers in epilepsy. Nat Rev Neurol. 2019;15(8):459–72. 31263255 10.1038/s41582-019-0217-x

[30] Zhang W, Du Y, Zou Y, Luo J, Lü Y, Yu W. Smad Anchor for Receptor Activation and Phospho-Smad3 Were Upregulated in Patients with Temporal Lobe Epilepsy. J Mol Neurosci. 2019;68(1):91–8. 30847724 10.1007/s12031-019-01285-0

[31] Paul D, Dixit A, Srivastava A, Tripathi M, Prakash D, Sarkar C, et al. Altered transforming growth factor beta/SMAD3 signalling in patients with hippocampal sclerosis. Epilepsy Res. 2018;146:144–50. 30153648 10.1016/j.eplepsyres.2018.08.004

[32] Kostoula C, Shaker T, Cerovic M, Craparotta I, Marchini S, Butti E, et al. TLR3 preconditioning induces anti-inflammatory and anti-ictogenic effects in mice mediated by the IRF3/IFN-β axis. Brain Behav Immun. 2019;81:598–607. 31336144 10.1016/j.bbi.2019.07.021

[33] Ma JH, Eo JC, Lee C, Hwang I, Choi J, Shin SJ, et al. Type I interferon signaling enhances kainic acid-induced seizure severity. bioRxiv. 2024;2024.11.13.623521.

[34] McElroy SL, Winham SJ, Cuellar-Barboza AB, Colby CL, Ho AMC, Sicotte H, et al. Bipolar disorder with binge eating behavior: a genome-wide association study implicates PRR5-ARHGAP8. Transl Psychiatry. 2018;8(1):40. 29391396 10.1038/s41398-017-0085-3 PMC5804024

[35] Lin Q, Li W, Li Y, Liu P, Zhang Y, Gong Q, et al. Aberrant structural rich club organization in temporal lobe epilepsy with focal to bilateral tonic-clonic seizures. Neuroimage Clin. 2023;40: 103536. 37944396 10.1016/j.nicl.2023.103536 PMC10663961

[36] Cheah PS, Prabhakar S, Yellen D, Beauchamp RL, Zhang X, Kasamatsu S, et al. Gene therapy for tuberous sclerosis complex type 2 in a mouse model by delivery of AAV9 encoding a condensed form of tuberin. Sci Adv. 2021;7(2):eabb1703. 33523984 10.1126/sciadv.abb1703 PMC7793581

[37] Franz DN, Krueger DA. mTOR inhibitor therapy as a disease modifying therapy for tuberous sclerosis complex. Am J Med Genet C Semin Med Genet. 2018;178(3):365–73. 30307123 10.1002/ajmg.c.31655

[38] Xiao L, Tang Y, Deng C, Li J, Li R, Zhu H, et al. Differences in whole-brain metabolism are associated with the expression of genes related to neurovascular unit integrity and synaptic plasticity in temporal lobe epilepsy. Eur J Nucl Med Mol Imaging. 2023;51(1):168–79. 37707571 10.1007/s00259-023-06433-8

[39] Li R, Xiao L, Han H, Long H, Liao W, Yang Z, et al. Transcriptionally downregulated GABAergic genes associated with synaptic density network dysfunction in temporal lobe epilepsy. Eur J Nucl Med Mol Imaging. 2025. 10.1007/s00259-024-07054-5. Online ahead of print 10.1007/s00259-024-07054-5 39777496

[40] Ballerini A, Biagioli N, Carbone C, Chiari A, Tondelli M, Vinceti G, et al. Late-onset temporal lobe epilepsy: insights from brain atrophy and Alzheimer’s disease biomarkers. Brain. 2024;25:awae207. 10.1093/brain/awae207 38915268

[41] Kaestner E, Reyes A, Chen A, Rao J, Macari AC, Choi JY, et al. Atrophy and cognitive profiles in older adults with temporal lobe epilepsy are similar to mild cognitive impairment. Brain. 2021;144(1):236–50. 33279986 10.1093/brain/awaa397 PMC7880670

